# Bacteria-specific modified nucleoside is released and elevated in urine of patients with bacterial infections

**DOI:** 10.1128/mbio.03124-24

**Published:** 2024-12-11

**Authors:** Ryosuke Yamamura, Yu Nagayoshi, Kayo Nishiguchi, Hitomi Kaneko, Keiichi Yamamoto, Koki Matsushita, Miho Shimamura, Akihiro Kunisawa, Korin Sakakida, Takeshi Chujo, Masataka Adachi, Yutaka Kakizoe, Yuichiro Izumi, Takashige Kuwabara, Masashi Mukoyama, Kazuhito Tomizawa

**Affiliations:** 1 Department of Molecular Physiology, Faculty of Life Sciences, Kumamoto University, Kumamoto, Japan; 2 Department of Nephrology, Faculty of Life Sciences, Kumamoto University, Kumamoto, Japan; 3 Center for Metabolic Regulation of Healthy Aging, Faculty of Life Science, Kumamoto University, Kumamoto, Japan; 4 Department of Laboratory Medicine, Kumamoto University Hospital 157728 , Kumamoto, Japan; 5 Department of Metabolic Medicine, Faculty of Life Sciences, Kumamoto University, Kumamoto, Japan; The Ohio State University School of Medicine, Columbus, Ohio, USA; The University of Texas at Dallas, Richardson, Texas, USA

**Keywords:** modified nucleoside, bacterial infection, biomarker, LC-MS, RNA modification

## Abstract

Over 170 types of chemical modifications have been identified in cellular RNAs across the three domains of life. Modified RNA is eventually degraded to constituent nucleosides, and in mammals, modified nucleosides are released into the extracellular space. By contrast, the fate of modified nucleosides in bacteria remains unknown. In this study, we performed liquid chromatography-mass spectroscopy (LC-MS) analysis of modified nucleosides from the RNA of 23 pathogenic bacteria, revealing 2-methyladenosine (m^2^A) as a common bacteria-specific modified nucleoside detected in all bacterial RNAs. Under normal culture conditions, bacteria did not actively release most modified nucleoside species, but robustly released nucleosides, including m^2^A, following addition of antibiotics or immune cells. These results indicate that m^2^A is released following bacterial lysis. Intraperitoneal injection of mice with m^2^A increased detectable levels of m^2^A in the urine, indicating that mammals can effectively excrete m^2^A. Additionally, mice infected with wild-type *E. coli* showed higher levels of m^2^A in their urine than mice infected by m^2^A-deficient *rlmN* KO *E. coli*. This suggests that m^2^A from the infected bacteria is excreted in the urine. Lastly, clinical studies using urine samples from febrile patients revealed significantly elevated levels of m^2^A during bacterial infections, and these values did not correlate with inflammation severity markers, such as white blood count (WBC) and C-reactive protein (CRP). This study reports the mammalian metabolism of modified nucleosides derived from bacterial RNA, and the elevation of urinary m^2^A in patients with bacterial infections.

**IMPORTANCE:**

This study reveals the differences in the fate and release of modified nucleosides in bacteria and mammals. Additionally, our study highlights that external bacteria-damaging factors, such as antibiotics and phagocytosis by host immune cells, promote the release of bacteria-specific modified nucleosides. Furthermore, we found that m^2^A was elevated in the urine from animal models of bacterial infection and the urine of patients with bacterial infections. Collectively, this work spans basic biology and clinical science, offering valuable insights into the fate of modified nucleosides in bacterial systems and their relevance to infectious diseases.

## INTRODUCTION

RNAs are critical to protein synthesis across the tree of life. RNAs contain over 170 types of post-transcriptional chemical modifications (1), which are essential to ensure the fidelity of protein synthesis and the stability of RNA structures (2). Due to the importance of RNA modifications, deficits in these modifications have been linked to various diseases in humans, including type 2 diabetes, mitochondrial myopathy, and intellectual disability (3–6).

Modified RNAs are eventually degraded into single nucleosides within the cytosol, and modified nucleosides are subsequently released from human cells into the extracellular space through equilibrative nucleoside transporters (ENTs) (7). Elevated levels of specific modified nucleosides in the blood and urine have been suggested as diagnostic biomarkers for the presence and severity of COVID-19 (8).

Among the diverse reported RNA modifications, some bacteria-specific modifications have been described (1), including 2-methyladenosine (m^2^A) and *N^4^*,2′-*O*-dimethylcytidine (m^4^Cm). A2503 is modified to m^2^A in 23S ribosomal RNA, as is position 37 of transfer RNAs (Arg^ACG^, Glu^UUC^, Asp^GUC^, His^GUG^, and Gln^UUG, CUG^) in *Escherichia coli* (9–11). m^2^A has also been reported in RNA species from *Bacillus subtilis*, *Mycobacterium smegmatis, Mycobacterium tuberculosis,* and *Staphylococcus aureus* (12–15). m^2^A modifications are catalyzed by a radical S-adenosylmethionine-dependent methyltransferase, RlmN, and deficits of this modification are associated with antibiotic resistance (12, 16–18). In the bacterial 16S rRNA decoding center, the cytosine at position 1402 in *E. coli* is converted to m^4^Cm by RsmH and RsmI, which is considered to increase translational fidelity (19). Despite advances in our understanding the writers and functions of RNA modifications, the metabolism of bacterial modified nucleosides following RNA degradation remains unclear.

In our study, we have identified that in contrast to mammals, bacteria do not release most modified nucleosides under normal physiological conditions. By contrast, m^2^A is detected in the extracellular space following antibiotic treatment or phagocytosis by immune cells. In addition, our clinical studies suggest that m^2^A in urine is elevated upon bacterial infection.

## MATERIALS AND METHODS

### Culture of pathogenic bacterial strains and RNA extraction

Pathogenic bacteria were isolated from patient samples at the Department of Laboratory Medicine, Kumamoto University Hospital, and the bacterial species were discerned using the VITEK MS system (bioMérieux Inc., Lyon, France). We used following strains: *Acinetobacter baumannii*, *Aeromonas hydrophila*, *Achromobacter xylosoxidans*, *Citrobacter freundii*, *Citrobacter koseri, Corynebacterium striatum*, *Enterobacter cloacae*, *Escherichia coli*, *Klebsiella aerogenes*, *Klebsiella oxytoca*, *Klebsiella pneumoniae*, *Morganella morganii*, *Pseudomonas aeruginosa*, *Proteus vulgaris*, *Raoultella ornithinolytica*, *Staphylococcus aureus, Staphylococcus caprae*, *Streptococcus dysgalactiae*, *Staphylococcus epidermidis*, *Staphylococcus haemolyticus*, *Staphylococcus hominis*, *Staphylococcus lugdunensis*, and *Stenotrophomonas maltophilia*. Pathogenic bacteria were cultured in LB Broth (Thermo Fisher Scientific, Waltham, MA, USA) for 24 h unless otherwise stated. We collected the culture medium of *A. baumannii*, *A. hydrophila*, *K. aerogenes*, *C. freundii*, *C. striatum*, *E. cloacae*, *P. aeruginosa*, *P. vulgaris*, *S. aureus, S. epidermidis*, *S. lugdunensis*, and *S. maltophilia* at 6, 12, 18, and 24 h. Bacterial pellets were obtained by centrifugation at 2,500 rpm for 15 min at 20°C (Model 7780 and RS-722G, Kubota Corporation, Tokyo, Japan). RNA was subsequently extracted using the TRI Reagent (Sigma Aldrich, St. Louis, MO, USA) or MORA-EXTRACT (KYOKUTO Pharmaceutical industrial, Tokyo, Japan). To obtain single nucleosides, total RNA was degraded using nuclease P1 and alkaline phosphatase.

### Culture of *Escherichia coli* (ATCC25922) and experimental treatments

*E. coli* (ATCC25922) was cultured in 5 mL of LB broth. Following 6, 12, 18, and 24 h of culture, the samples were centrifuged at 2,500 rpm for 15 min at 20°C (Model 7780 and RS-722G, Kubota Corporation, Tokyo, Japan). The medium and bacterial lysate generated using RIPA buffer (50 mM Tris-HCl [pH 8.0], 150 mM NaCl, 1% NP-40, 0.5% sodium deoxycholate, 0.1% sodium dodecyl sulfate, and protease inhibitor [Roche, Basel, Switzerland]) were collected for liquid chromatography-mass spectroscopy (LC-MS) analysis. Antibiotic treatment experiments were performed by adding kanamycin (50 µg/mL), clarithromycin (8 µg/mL), and chloramphenicol (25 µg/mL) to the medium for 24 h.

### LC-MS sample preparation and analysis

Samples were desalted and deproteinized using a Nanosep 3K Omega (Pall Corporation, New York, NY, USA) by centrifugation at 12,000 rpm at 4°C for 30 min (Model 3740 and AF2724A, Kubota Corporation, Tokyo, Japan). Modified nucleoside quantification was performed using a triple quadrupole mass spectrometry system (LCMS-8060NX, Shimadzu Corporation, Kyoto, Japan) equipped with an electrospray ionization (ESI) source and an ultra-high performance liquid chromatography system (7, 8, 20). Samples were injected into an Inertsil ODS-3 column (GL Science, Tokyo, Japan). The mobile phase consisted of two types of solutions: 5 mM ammonium acetate in water adjusted to pH 5.3, and 60% (vol/vol) acetonitrile in water. The LC gradient was established as follows: 1–10 min: 1%–22.1% B, 10–15 min: 22.1%–63.1% B, 15–17 min: 63.1%–100% B, 17–22 min: 100% B, and 22–23 min, 100%–0.6% B. The flow rate was 0.4 mL/min, and the injection volume was 2 µL. Detection was performed in the multiple reaction monitoring (MRM) modes in the LabSolutions System (Shimadzu Corporation). MRM transition parameters for modified nucleosides in this method are listed in Table S1. The interface temperature was 300°C, the desolvation line temperature was 250°C, and the heat block temperature was 400°C. Gaseous nitrogen was supplied from an N2 feeder Model T24FD (System Instruments, Tokyo, Japan) for nebulization and drying, and gaseous argon was used for collision-induced dissociation. The standards for detection of modified nucleosides are listed in Table S2.

### Mammalian cell culture, treatments, and RT-PCR

THP-1 cells (KAC Co., Ltd, Kyoto, Japan) were maintained in RPMI medium with 10% fetal bovine serum (FBS) and 2 mM L-glutamine (FUJIFILM Wako Chemical Corp, Osaka, Japan). THP-1 cells were differentiated using 1 µg/mL phorbol 12-myristate 13-acetate (PMA) (Adipogen Life Sciences, San Diego, CA, USA) for 72 h. HeLa and HEK293 cells were maintained in DMEM with 10% FBS. Gene knockdown of differentiated THP-1 cells was performed using the HiPerFect Transfection Reagent (QIAGEN, Venlo, Netherlands) using siRNAs listed in Table S3. RNA extraction was performed using TRI Reagent (Sigma Aldrich, St. Louis, MO, USA), and cDNA was synthesized with the PrimeScript RT reagent Kit (Takara Bio, Kusatsu, Japan). RT-PCR was performed with SYBR Green reagents (Takara Bio, Kusatsu, Japan) with primer sets listed in Table S3. Both THP-1 and HeLa cells were treated with 10 µg/mL m^2^A. Cell growth was measured using the Cell Counting Kit-8 (Dojindo, Kumamoto, Japan).

### Co-culture of mammalian cell lines with *Escherichia coli*

Co-culture experiments were performed with *E. coli* (ATCC25922) cultured in LB and differentiated THP-1 cells (21). THP-1 cells were seeded at 1 × 10^6^ cells/mL in 12-well tissue culture dishes and differentiated with PMA. THP-1 cells were infected with *E. coli* (ATCC25922, counted using hemocytometer) at a multiplicity of infection (MOI) of 10. The plate was centrifuged for 5 min at 1,000 × *g* (S700FR and RS-7504M, Kubota Corporation, Tokyo, Japan) to synchronize infection. After 20 min of incubation, the plates were washed with PBS and provided with fresh RPMI +10% FBS supplemented with 100 µg/mL of gentamicin (FUJIFILM Wako Chemical Corp, Osaka, Japan) to kill extracellular bacteria. After 2 h, the medium was replaced with fresh RPMI. After an additional 2 h incubation, the medium was collected for analysis.

### Mouse experiments

C57BL/6J mice were housed at 25°C with 12 h light and 12 h dark cycles. Male mice of 18 to 22 weeks of age were used for experiments. Chemically synthesized m^2^A was diluted in physiological saline to a concentration of 2 µM m^2^A, and then injected intraperitoneally at a volume of 10 µL/g (body weight). For infection experiments, *E. coli* (ATCC25922) or *rlmN* KO *E. coli* was cultured in LB broth, and then 8 × 10^5^/100 µL was injected into mice. Cefoperazon was diluted in autoclaved water to 0.5 mg/mL and administered orally from 1 day after bacterial injection until the end of the experiment via a drinking water bottle. Urine was collected in a metabolic cage.

### Collection of urine samples from patients

We enrolled patients presenting with fever in the Department of Nephrology, Kumamoto University Hospital, or Amakusa Medical Hospital. Patients were diagnosed with infection, or alternative cause of fever by clinical investigators using several methods, including blood culture and a pneumococcal urinary antigen test. Information pertaining to these patients is described in Tables S4 and S5.

### Statistical analysis

Data displaying a normal distribution and homogenous variance were expressed as the mean ± standard error of means (SEM) and were compared using the Mann–Whitney *U* or Wilcoxon signed-rank test. Categorical variables were compared using an ordinary one-way ANOVA with Dunnett’s multiple comparison test or the Kruskal–Wallis test followed by Dunn’s multiple comparison. Statistical analyses were performed using Prism 10 software (GraphPad, San Diego, CA, USA), and *P*-values less than 0.05 were considered statistically significant. For receiver operating characteristic (ROC) analysis, sensitivity and specificity at each cutoff value were calculated, followed by calculation of the likelihood ratio. The highest likelihood ratio was selected, and the corresponding cutoff value was set.

## RESULTS

### Detection of modified nucleosides in bacterial RNA by LC-MS

**Fig 1:**
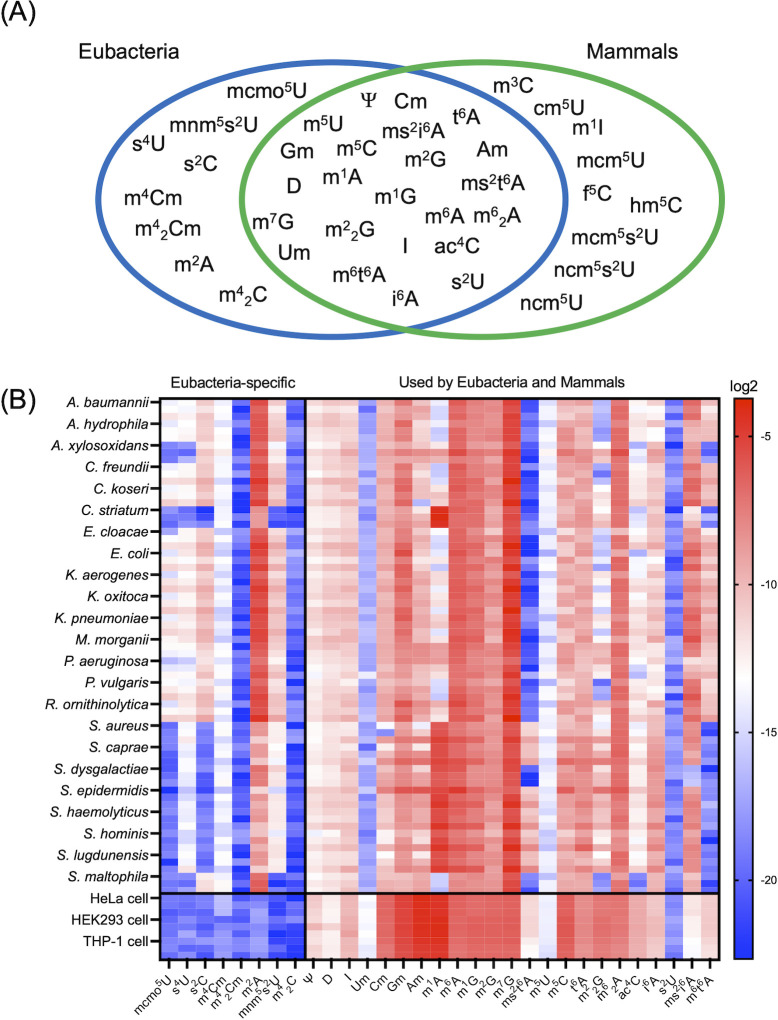
Profiling RNA modifications in 23 bacterial species and three mammalian cell lines. (**A**) Venn diagram classified by use of each modified nucleoside by eubacteria and mammals. mcmo^5^U: 5-methoxycarbonylmethoxyuridine, ms^2^i^6^A: 2-methylthio-*N^6^*-isopentenyladenosine, s^4^U: 4-thiouridine, s^2^C: 2-thiocytidine, m^3^U: 3-methyluridine, m^4^Cm: *N^4^*,2′-*O*-dimethylcytidine, m^4^_2_cm: *N^4^*,*N^4^*,2′-*O*-trimethylcytidine, m^2^A: 2-methyladenosine, m^6^t^6^A: *N^6^*-methyl-*N^6^*-threonylcarbamoyladenosine, m^4^_2_C: *N^4^*,*N^4^*-dimethylcytidine, Ψ: pseudouridine, Cm: 2′-*O*-methylcytidine, m^5^U: 5-methyluridine, m^5^C: 5-methylcytidine, t^6^A: *N^6^*-threonylcarbamoyladenosine, Am: 2′-*O*-methyladenosine, Gm: 2′-*O*-methylguanosine, mnm^5^s^2^U: 5-methylaminomethyl-2-thiouridine, m^1^A: *N^1^*-methyladenosine, m^6^A: *N^6^*-methyladenosine, D: dihydrouridine, m^1^G: *N^1^*-methylguanosine, m^2^G: *N^2^*-methylguanosine, m^7^G: 7-methylguanosine, m^2^_2_G: *N^2^*,*N^2^*-dimethylguanosine, m^6^_2_A: *N^6^*,*N^6^*-dimethyladenosine, I: inosine, Um: 2′-*O*-methyluridine, i^6^A: *N^6^*-isopentenyladenosine, ac^4^C: *N^4^*-acetylcytidine, s^2^U: 2-thiouridine, m^3^C: 3-methylcytidine, cm^5^U: 5-carboxymethyluridine, m^1^I: *N^1^*-methylinosine, mcm^5^U: 5-methoxycarbonylmethyluridine, f^5^C: 5-formylcytidine, hm^5^C: 5-hydroxymethylcytidine, mcm^5^s^2^U: 5-methoxycarbonylmethyl-2-thiouridine, ncm^5^s^2^U: 5-carbamoylmethyl-2-thiouridine, and ncm^5^U: 5-carbamoylmethyluridine. (**B**) Heatmap of RNA modification levels from 23 bacterial species and three mammalian cell lines. LC-MS peak areas of modified nucleosides divided by unmodified nucleosides are shown. The color scale indicates the auto-scaled relative mean of three biological replicates. *A. baumannii: Acinetobacter baumannii*, *A. hydrophila: Aeromonas hydrophila*, *A. xylosoxidans: Achromobacter xylosoxidans*, *C. freundii: Citrobacter freundii*, *C. koseri: Citrobacter koseri, C. striatum: Corynebacterium striatum*, *E. cloacae: Enterobacter cloacae*, *E. coli: Escherichia coli*, *K. aerogenes: Klebsiella aerogenes*, *K. oxitoca: Klebsiella oxytoca*, *K. pneumoniae: Klebsiella pneumoniae*, *M. morganii: Morganella morganii*, *P. aeruginosa: Pseudomonas aeruginosa*, *P. vulgaris: Proteus vulgaris*, *R. ornithinolytica: Raoultella ornithinolytica*, *S. aureus: Staphylococcus aureus, S. caprae: Staphylococcus caprae*, *S. dysgalactiae: Streptococcus dysgalactiae*, *S. epidermidis: Staphylococcus epidermidis*, *S. haemolyticus: Staphylococcus haemolyticus*, *S. hominis: Staphylococcus hominis*, *S. lugdunensis: Staphylococcus lugdunensis*, *S. maltophila: Stenotrophomonas maltophilia*.

To detect and quantify modified nucleosides in bacterial and human RNA using LC-MS, we discerned the corresponding peaks using chemically synthesized modified nucleosides (Table S1; Fig. S1). Using the MODOMICS database (1) and data from previous studies (2, 4, 22), we classified detectable modified nucleosides into three categories: eight eubacteria-specific modified nucleosides, nine mammal-specific modified nucleosides, and 23 commonly detected modified nucleosides (Fig. 1A). Next, we collected 23 Gram-positive and Gram-negative pathogenic bacterial strains. Total cellular RNA was extracted from each strain, and samples were digested into single nucleosides before analysis using LC-MS. We also measured the modified nucleoside constituents of RNA extracted from three human cell lines: HeLa cells, HEK293 cells, and differentiated THP-1 cells. Comparison of these data sets indicated that 2-methyladenosine (m^2^A) was robustly detected from all bacterial strains but not from mammalian cells (Fig. 1B). Other previously reported bacteria-specific modified nucleosides were not detected in mammalian samples and were detected in only a few bacterial samples. Some previously described modified uridine and cytidine species were not detected in our analysis. This may be due to their low abundance and/or low LC-MS ionization efficiency due to fewer nitrogen atoms in pyrimidine bases to which protons bind during LC-MS ionization.

From the resulting data set, we confirmed that eight of the monitored modified nucleosides were bacteria-specific: m^2^A, 5-methoxycarbonylmethoxyuridine (mcmo^5^U), 5-methylaminomethyl-2-thiouridine (mnm^5^s^2^U), 4-thiouridine (s^4^U), 2-thiocytidine (s^2^C), *N^4^*,*N^4^*,2′-*O*-trimethylcytidine (m^4^_2_Cm), *N^4^*,2′-*O*-dimethylcytidine (m^4^Cm), and *N^4^*,*N^4^*,dimethylcytidine (m^4^_2_C).

### Bacteria do not release most modified nucleosides species under physiological growth conditions

**Fig 2:**
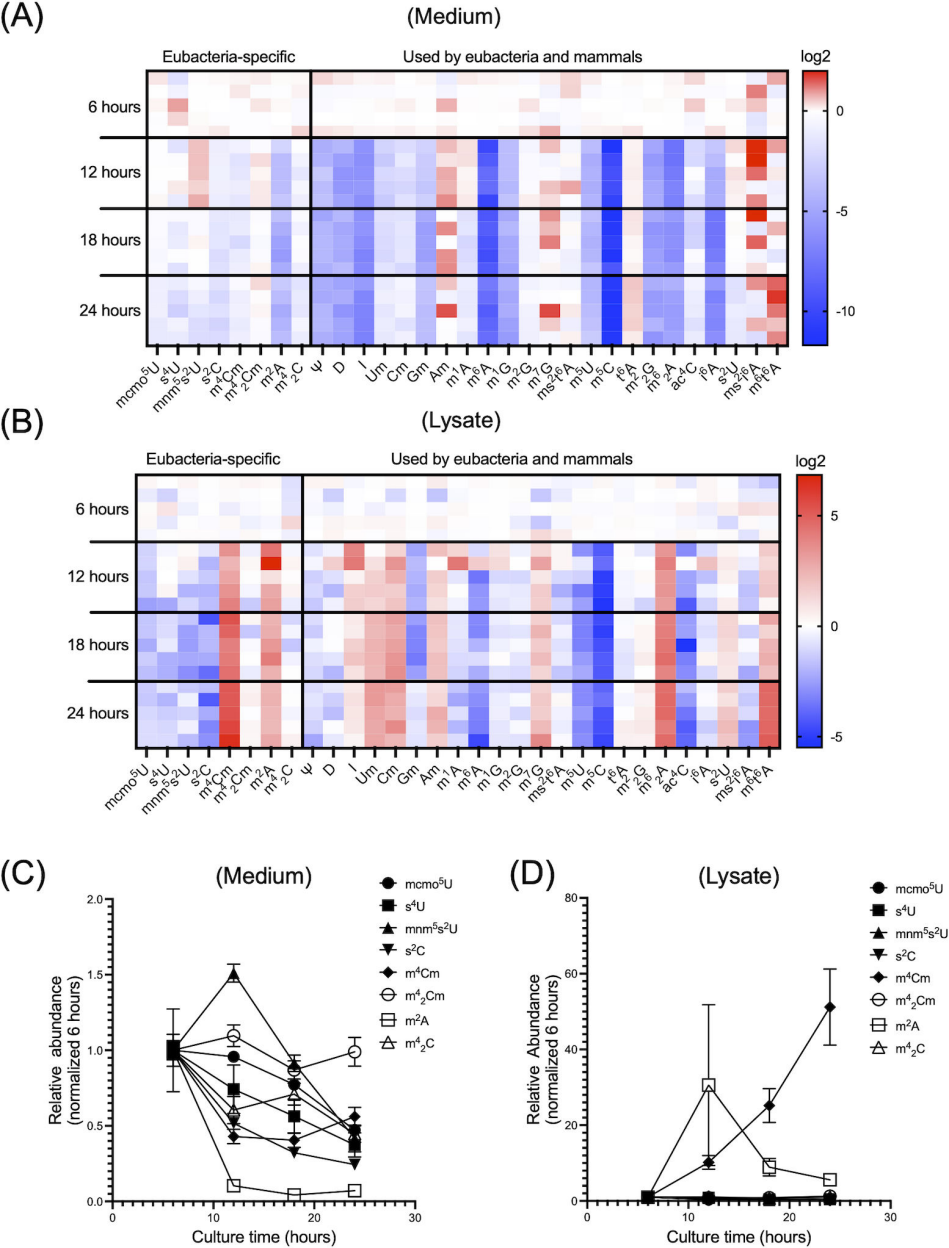
Modified nucleosides in the culture medium decrease during bacterial growth. (**A, C**) Heatmap and chart analysis of modified nucleoside levels in the culture medium from *E. coli* (ATCC25922) over time. The color scale indicates the auto-scaled relative mean of four biological replicates. Abbreviations refer to Fig. 1A. (**B, D**) Heatmap and chart analysis of modified nucleoside levels in *E. coli* (ATCC25922) lysates at each time point. The color scale shows the auto-scaled relative mean of four biological replicates. Abbreviations refer to Fig. 1A.

We previously reported that modified nucleosides were excreted into extracellular spaces by mammalian cells (7). Prior to the present study, the fate of modified nucleosides following RNA degradation in bacteria was unclear. We performed nucleoside analysis of free nucleoside monomers derived from cellular RNA metabolism (and not the modified nucleosides within RNA molecules). First, we quantified modified nucleosides in culture medium during *E. coli* (ATCC25922) growth. During culture, the concentration of most initially detected modified nucleoside species, which likely derive from the medium, decreased (Fig. 2A and C). At 12 h, mnm^5^s^2^U transiently increased and subsequently decreased (Fig. 2A and C). We also performed the same culture experiments using 12 other bacterial species (Fig. S2), which showed that most bacteria-specific modified nucleosides were not released under normal culture conditions from these bacterial species. One exception was m^4^Cm, which was released by many strains during the course of culture (Fig. S2). Next, we collected *E. coli* cells and measured free modified nucleosides in lysates. The concentrations of several nucleosides, including m^2^A and m^4^Cm, increased in the lysates over time (Fig. 2B and D). These results suggest that *E. coli* did not actively release most modified nucleosides into the extracellular space under normal growth conditions, but accumulated m^2^A and m^4^Cm intracellularly. The metabolism of modified nucleosides therefore differs between bacteria and mammals.

### Bacteria-specific modified nucleosides are released following bacterial lysis induced by antibiotics or immune cells

**Fig 3:**
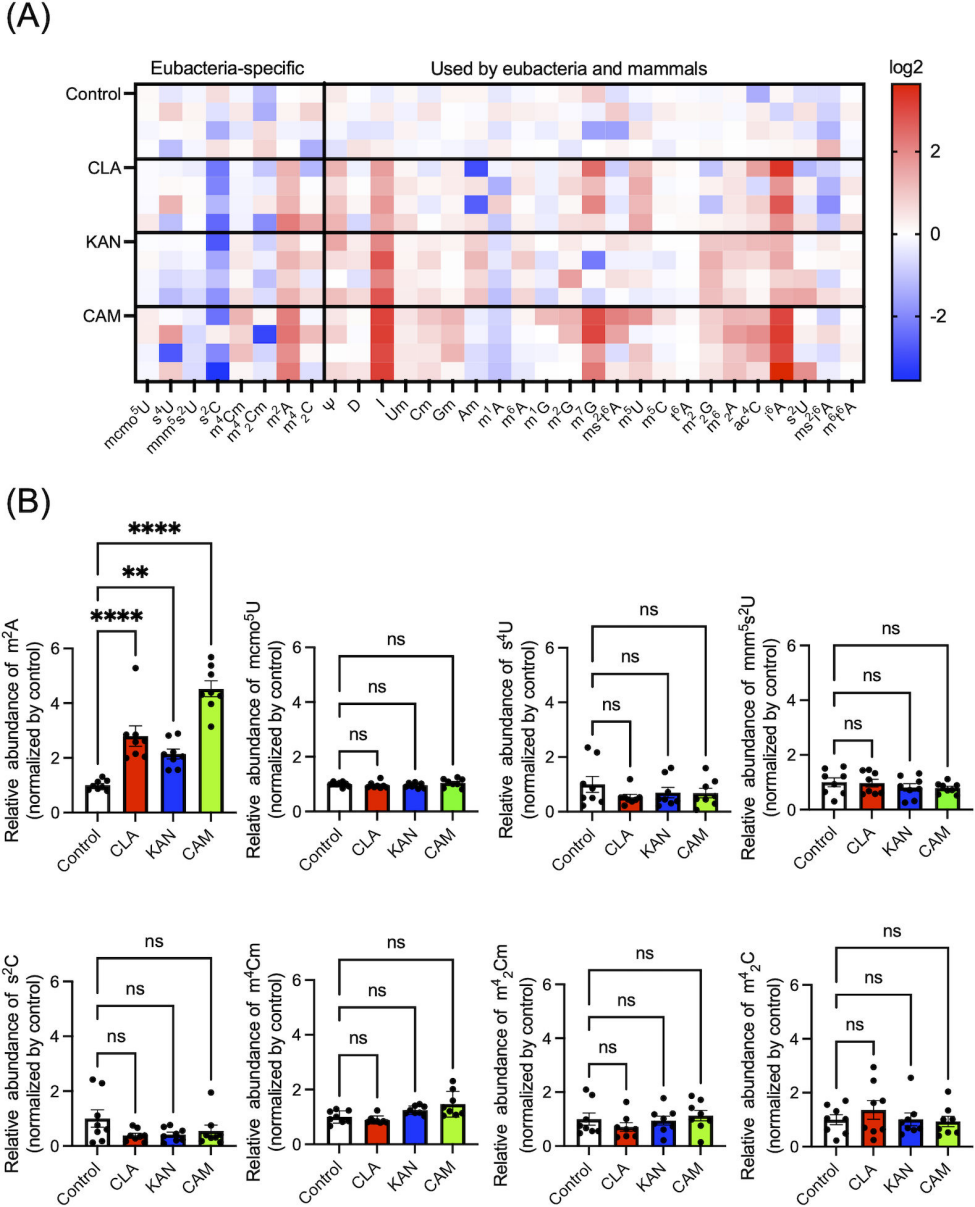
Modified nucleosides are secreted following treatment with bactericidal antibiotics. (**A**) Heatmap analysis of modified nucleoside levels in *E. coli* (ATCC25922) culture medium following treatment with antibiotics. CLA: clarithromycin, KAN: kanamycin, CAM: chloramphenicol. The color scale indicates the auto-scaled relative mean of four biological replicates. Abbreviations in the heatmap refer to Fig. 1A. (**B**) Measurement of bacteria-specific modified nucleosides in the culture medium. **: *P* < 0.01, ****: *P* < 0.0001, ns: not significant. Data were analyzed using a one-way ANOVA and Dunnett’s multiple comparison test.

Next, we hypothesized that m^2^A would be released when bacteria were exposed to lytic stimuli. To investigate this hypothesis, we first administered antibiotics to the culture medium of *E. coli* (ATCC25922) and quantified the release of modified nucleosides. We observed an increase of several modified nucleosides when cultures were treated with the antibiotics, clarithromycin (CLA), kanamycin (KAN), and chloramphenicol (CAM) (Fig. 3A). In particular, concentrations of the bacteria-specific modified nucleoside, m^2^A, significantly increased in the culture medium of *E. coli* (ATCC25922) (Fig. 3B).

**Fig 4:**
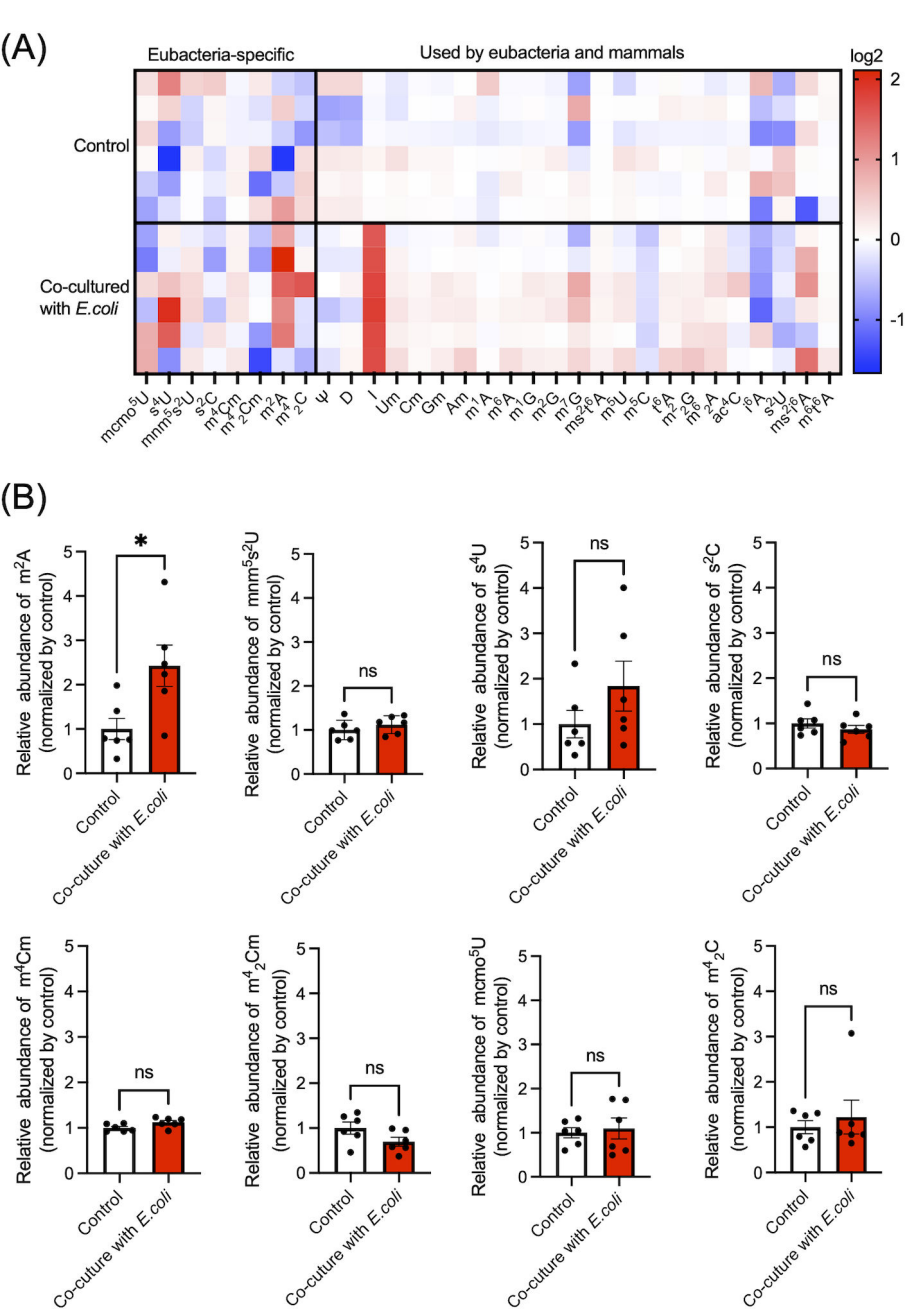
Modified nucleosides are released following phagocytosis by differentiated THP-1 cells. (**A**) Heatmap analysis of modified nucleoside levels in the culture medium of THP-1 cells co-cultured with *E. coli* (ATCC25922). The color scale indicates the auto-scaled relative mean of six biological replicates. (**B**) Measurements of bacteria-specific modified nucleosides in culture medium. *: *P* < 0.05. Data were analyzed using a Mann–Whitney test.

We also examined alterations of modified nucleoside release under conditions where bacteria are damaged by human monocytes. We co-cultured the representative monocyte cell line, THP-1, and *E. coli* (ATCC25922) before collecting culture medium for analysis by LC-MS. After co-culturing, gentamicin was added to the medium to kill bacteria that had not been phagocytosed by THP-1 cells. THP-1 cells were then transferred to new medium and incubated for 2 h, and the medium was examined to determine whether it contained bacteria-specific modified nucleosides derived from the bacteria phagocytosed by THP-1 (Fig. 4A). We compared the results of co-cultured medium with a control THP-1 sample supplemented with LB medium but no bacteria (Fig. 4A). We observed that the levels of several modified nucleosides were elevated in the co-culture medium but not in the control. m^2^A was the only bacteria-specific nucleoside that significantly increased following co-culture with *E. coli* (Fig. 4B). In mammals, equilibrium nucleoside transporters (ENTs) are responsible for the excretion of unmodified nucleosides (23). In addition, modified nucleosides are also excreted extracellularly by ENT1 and ENT2 in mammals (7). To examine the importance of ENTs for release of bacteria-specific modified nucleosides, we genetically downregulated the expression of each ENT in differentiated THP-1 cells using siRNA (Fig. S3) before co-culturing these cells with *E. coli* (ATCC25922). Knockdown of ENTs did not affect the release of bacteria-specific modified nucleosides (Fig. S4).

Recently, it was reported that single nucleosides activate Toll-like receptors in endosomes (24, 25). Further, activation of adenosine receptors affects cell growth, and we have previously reported that *N*^6^-methyladenosine stimulates the adenosine A3 receptor better than unmodified adenosine (20, 26–29). To investigate if m^2^A may stimulate the innate immune response or cell growth, we treated differentiated THP-1 cells with chemically synthesized m^2^A and evaluated the mRNA expression of *IL-6*, *TNF-α*, and *IL-15*. We observed that treatment with m^2^A did not stimulate immune gene expression (Fig. S5). We also observed that treatment of HeLa cells with m^2^A did not affect growth (Fig. S6).

Collectively, these results demonstrate that several bacteria-derived modified nucleosides, including m^2^A, are released upon bacterial damage by antibiotic treatment or immune cell exposure.

### Extracellular m^2^A is excreted into urine, and urine m^2^A level elevates upon bacterial infection in mice

**Fig 5:**
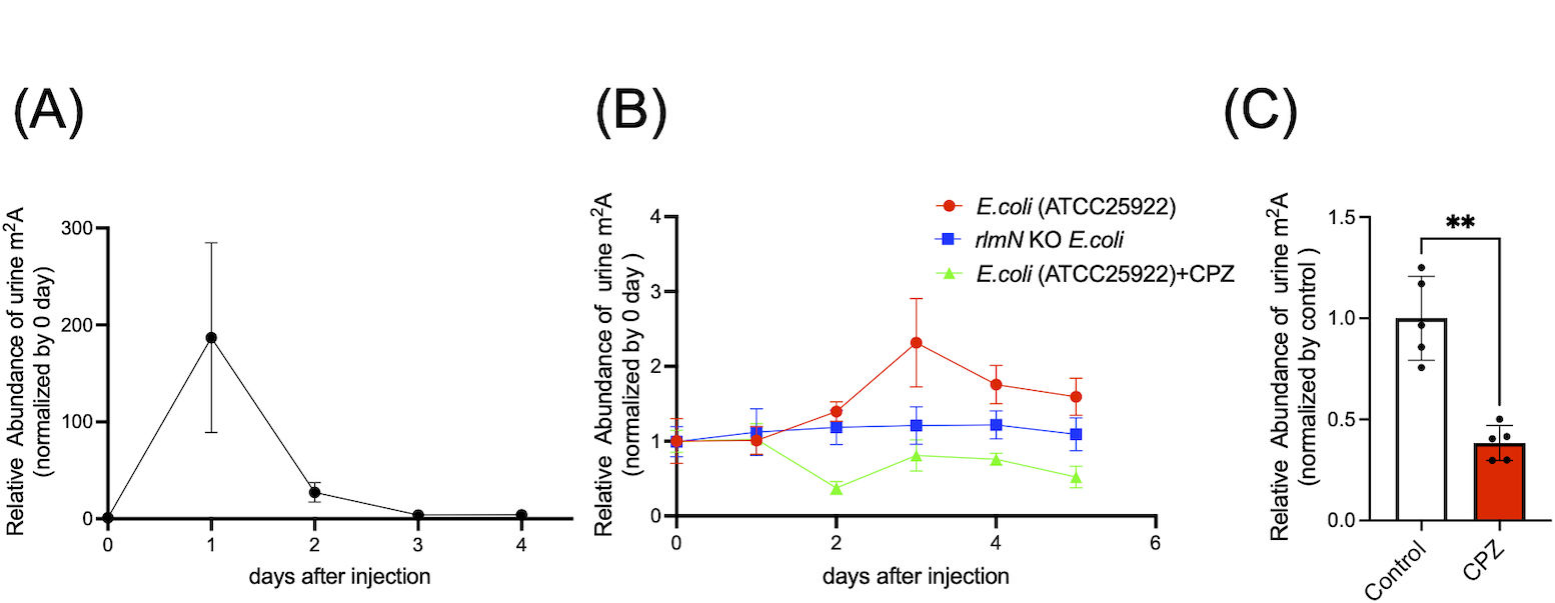
Measurement of m^2^A levels in the urine of mice after intraperitoneal injection of synthetized m^2^A or infection with bacteria. (**A**) Measurement of m^2^A levels in the urine of mice after intraperitoneal injection of m^2^A. (**B**) Measurement of m^2^A levels in the urine of mice after intraperitoneal injection of *E. coli* (ATCC25922) or *rlmN* KO *E. coli*. *n* = 4; CPZ, mice orally administered with cefoperazone. (**C**) Measurement of m^2^A levels in the urine of mice 1 week after administration of CPZ. *n* = 4, **: *P* < 0.01. Data were analyzed using Mann–Whitney test.

We previously reported that in mammals, many modified nucleosides are excreted via the urine (20). We therefore hypothesized that bacteria-derived modified nucleosides, including bacteria-specific m^2^A, may also be excreted via urine following release from immune cells after bacterial phagocytosis. Based on its robust detection by measurements of LC-MS/MS, we selected m^2^A as the bacteria-specific modified nucleoside for study. We first intraperitoneally injected mice with m^2^A and monitored m^2^A levels in the urine. We observed that 1 day after m^2^A injection, m^2^A increased approximately 200-fold in the urine compared with basal levels. Concentrations subsequently rapidly decreased by day 2 and were almost at basal levels by day 3 (Fig. 5A). We next performed infection experiments in mice by injecting *E. coli* (ATCC25922) or *rlmN* KO *E. coli* intraperitoneally. Three days after injection, the urine level of m^2^A increased in mice infected with *E. coli* (ATCC25922) (Fig. 5B, red circles) but not in mice infected with *rlmN* KO *E. coli*. (Fig. 5B, blue squares). This result strongly suggested that m^2^A derived from infected *E. coli* (ATCC25922) was the source of the increased m^2^A in mouse urine. To further confirm that m^2^A was derived from bacteria, 1 day after infection, we orally administered cefoperazone (CPZ) to mice every day via drinking water and collected urine. The m^2^A level did not increase after intraperitoneal bacterial administration when the mice were treated with CPZ (Fig. 5B, light green triangles). During these experiments, we observed that unlike mammalian cellular RNA (Fig. 1B), mouse urine always contained a basal level of “bacteria-specific” modified nucleosides. We hypothesized that such “bacteria-specific” nucleosides in healthy mouse urine might be derived from intestinal microbiota. To investigate this hypothesis, we examined the effect of antibiotics on m^2^A excretion by orally administering CPZ to healthy mice. As a result, we observed that the urine m^2^A level decreased after 1 week treatment with CPZ (Fig. 5C), suggesting that the basal m^2^A level in urine was derived from bacteria within the mouse body, presumably those that had been absorbed via the intestine. Compared with the m^2^A level after *in vitro* antibiotic treatment (Fig. 3), the urine m^2^A level of mice treated with CPZ (Fig. 5B, green triangles) was not acutely enhanced. This might be partly because, compared with intestinal bacteria living under a relatively anaerobic environment, cultured bacteria living under an oxygen-rich, nutrient-rich condition rapidly propagate and thus might be more sensitive to antibiotics. However, further work will be required to confirm this conjecture.

### Urine m^2^A level is elevated upon bacterial infection in human

**Fig 6:**
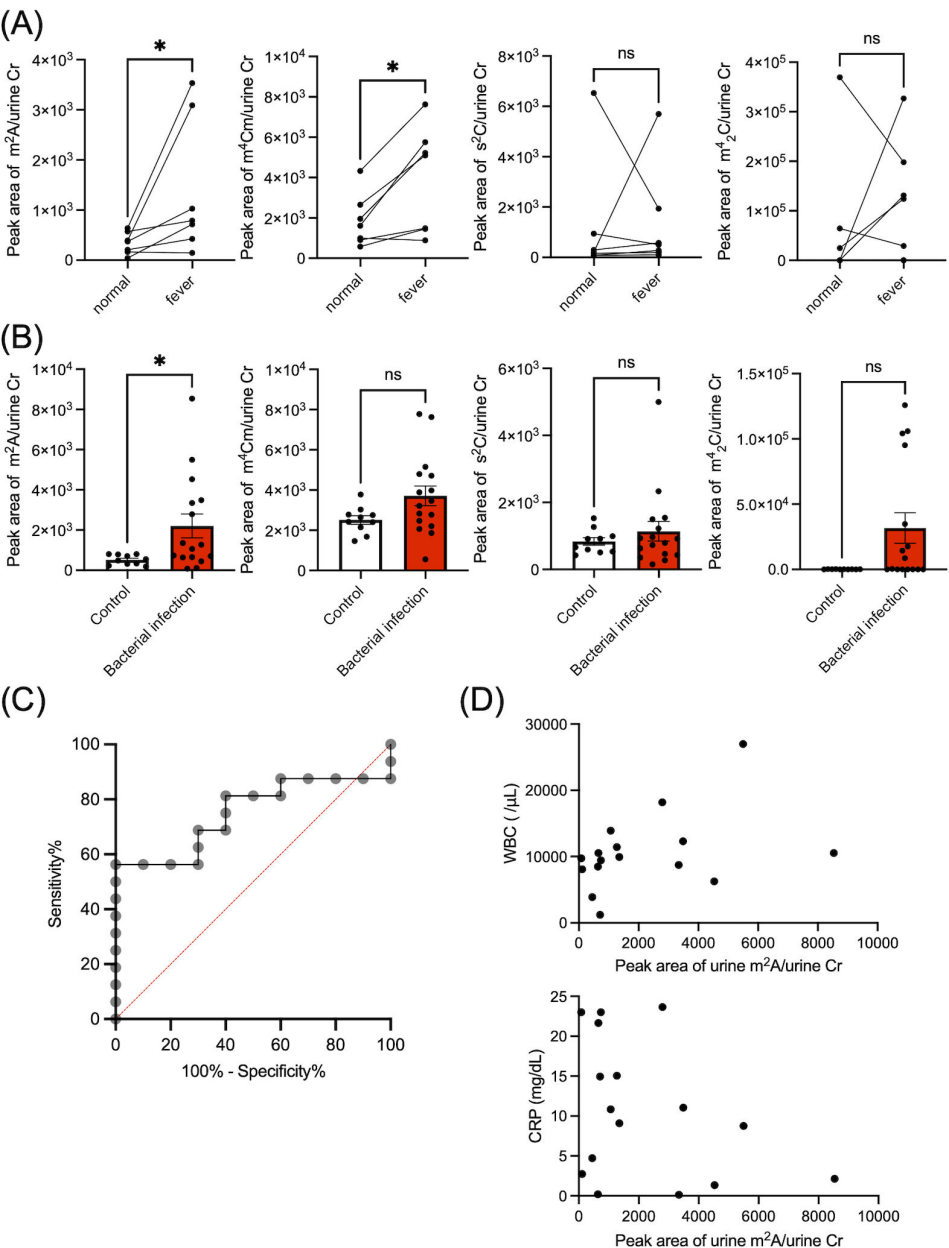
m^2^A level in the urine as a potential biomarker of bacterial infection. (**A**) Measurements of bacteria-specific modified nucleosides in the urine of patients with bacterial infection at normal phase and febrile phase. LC-MS peak areas of modified nucleosides divided by urine creatinine are shown. *: *P* < 0.05. Data were analyzed using the Wilcoxon signed-rank test. (**B**) Measurements of bacteria-specific modified nucleosides in the urine of patients with bacterial infection or healthy volunteers. LC-MS peak areas of modified nucleosides divided by urine creatinine are shown. *: *P* < 0.05. Data were analyzed using the Wilcoxon signed-rank test. (**C**) ROC analysis of m^2^A levels in urine normalized by urine creatinine was performed for calculation of sensitivity and specificity. (**D**) Correlation of m^2^A levels in the urine normalized by urine creatinine and WBC or CRP values.

We next analyzed bacteria-specific modified nucleoside levels in the urine of patients with fever. Patients were categorized into two groups following clinical examination: the bacterial infection group and the non-bacterial infection group (Table S4). As urine composition is highly susceptible to host physiological conditions (e.g., water intake), continuous urine collection over a period of 1 day is preferred for the quantitative evaluation of biomarkers. However, in clinical practice, continuous urine collection over a period of 1 day is difficult to perform because it requires hospitalization and lowers patients’ quality of life (QOL). Thus, considering clinical feasibility, urine creatinine has been used for physiological normalization of spot urine collected at the bedside (30, 31). We compared levels of modified nucleosides in the urine normalized by urine creatinine levels, between the fever phase and normal phase of each group. This revealed that levels of m^2^A and m^4^Cm were significantly increased in the fever phase of the bacterial infection group, but not in the non-infection group (Fig. 6A; Fig. S7). Instead, s^2^C levels were increased during the fever phase only in the non-bacterial infection group (Fig. S7). We next compared the urine of healthy controls and patients with bacterial infection (Table S5). m^2^A levels were significantly elevated in the urine of bacterial infection patients (Fig. 6B). Basal levels of bacteria-specific modified nucleosides were also detected in healthy controls (Fig. 6B, Control), which presumably originated from the intestinal microbiota, as suggested by the results of healthy mice (Fig. 5C). LC-MS analysis revealed that other bacteria-specific modified nucleosides, i.e., mnm^5^s^2^U, s^4^U, m^4^_2_Cm, and mcmo^5^U, were undetectable, likely due to the presence of substances in urine that suppress LC-MS ionization efficiency. We also performed receiver operating characteristic (ROC) analysis. ROC analysis revealed that considering a cutoff value of 592.8 for m^2^A in urine, our analysis had a sensitivity of 81.3% and a specificity of 60% (Fig. 6C). Further analysis of patient-associated clinical data indicated no correlation between m^2^A levels in urine and WBC and CRP scores as proxies of inflammation (Fig. 6D).

Therefore, the elevated levels of m^2^A in patient urine were not associated with the severity of inflammation, but rather the presence of bacterial infection.

## DISCUSSION

In this study, we first confirmed that eight types of monitored modified nucleosides are specific to bacterial cells. m^2^A is widely present in both Gram-positive and negative bacteria, but not in mammals. Indeed, previous studies have described m^2^A in *E. coli*, *B. subtilis*, *M. smegmatis, M. tuberculosis,* and *S. aureus*. (9–15). While our analysis could quantify multiple diverse modified nucleosides, we were unable to quantify some modified nucleosides due to lack of synthetic nucleosides for comparison, for example, bacteria-specific lysidine (32–34). Analysis of additional nucleosides will be the focus of future studies by our group.

Next, we found that most modified nucleosides were not actively exported under normal growth conditions in *E. coli*. Our previous studies in mammals revealed that modified nucleosides generated through RNA degradation are actively exported to the extracellular space by ENTs 1 and 2 (7). By contrast, in *E. coli*, most extracellular modified nucleosides decreased during normal bacterial growth (Fig. 2A and C). Twelve other bacterial species also did not release most bacteria-specific modified nucleosides under normal culture condition; however, many strains released m^4^Cm (Fig. S2). m^4^Cm was also present in the lysates of *E. coli* and increased in the urine of patients during the febrile phase of bacterial infections (Fig. 2D and 6A). These results suggest that unlike other modified nucleosides, m^4^Cm might not be easily “recycled” in bacteria. Further study will be required to test this hypothesis. Although we found that most modified nucleosides were not actively exported under normal growth conditions, the intracellular fate of modified nucleosides under physiological growth conditions still remains unclear and will be a focus of future studies.

We also examined whether release of modified nucleosides would be influenced by external factors. We found that in cultured *E. coli*, antibiotic treatment promoted release of various modified nucleosides (Fig. 3A). m^2^A was the only bacteria-specific modified nucleoside that was clearly elevated in the extracellular medium under these conditions (Fig. 3B). These data suggest that m^2^A is released when bacteria are damaged by antibiotics. Interestingly, previous studies have reported that m^2^A and similar derivatives possess antimycobacterial activity (35).

In mammals, the immune system responds to bacterial infection. We therefore co-cultured human macrophages with *E. coli* and found that the concentration of several modified nucleosides increased in the cell culture medium (Fig. 4A), and m^2^A was the only significantly increased bacteria-specific nucleoside (Fig. 4B). Following phagocytosis, bacteria are digested in lysosomal compartments, and bacterial RNA is subsequently degraded by RNases (36). In the phagolysosome, nucleosides and bacterial RNA fragments stimulate innate immune responses *via* endosomal-derived Toll-like receptor 8 (36, 37). Toll-like receptor eight recognizes single uridine as the primary ligand, and then cytidine metabolites (38, 39). In our model, however, treatment of THP-1 cells with m^2^A did not activate immune gene expression (Fig. S4). These data indicate that extracellular release of m^2^A is specific to bacterial damage and is not associated with an immune response. We next tried to examine the mechanism by which bacteria-specific modified nucleosides are released in mammalian cells. Knockdown of each equilibrative nucleoside transporter (Fig. S3 and S4) indicated that these proteins may not be involved in the release of bacteria- specific modified nucleosides. To definitively confirm a role of host transporters in our phenotype, future experiments will focus on genetic deletion of ENTs and other transporters such as concentrative nucleoside transporter (40).

We previously reported that modified nucleosides in mammals were finally excreted into the urine (20). We therefore hypothesized that bacteria-derived modified nucleosides are also excreted *via* the urine. Indeed, upon intraperitoneal injection of mice with chemically synthesized m^2^A, most m^2^A was excreted into the urine within 48 h (Fig. 5A). We also examined the urine of mice infected by intraperitoneal injection of *E. coli* (ATCC25922) or m^2^A-deficient *rlmN* KO *E. coli* to mice (Fig. 5B) and demonstrated that m^2^A derived from the infected bacteria was excreted in their urine. Moreover, we observed antibiotic administration decreased m^2^A excretion significantly (Fig. 5C).

Next, we demonstrated that m^2^A levels were significantly enriched in the urine of febrile patients with bacterial infections, but not in febrile patients with non-bacterial etiology or in healthy volunteers (Fig. 6A and B; Fig. S7). However, there are two limitations in using m^2^A to detect bacterial infection in humans. First, specificity of using m^2^A in urine for bacterial infection is insufficient for clinical use (Fig. 6C). Second, m^2^A is used universally by bacterial strains; hence, the elevation of m^2^A in urine cannot differentiate the type of bacteria causing infection. Comparison of urine m^2^A levels with WBC or CRP levels, which are common markers of infection severity and inflammation, indicated no significant correlation (Fig. 6D). Therefore, elevated m^2^A in the urine was not caused by the severity of the inflammation, but by the presence or absence of infectious bacteria. In such a future study, improvements may be needed, such as combining analysis of m^2^A with (1) other bacterial modified nucleosides like m^4^Cm and uninvestigated bacterial modified nucleosides like lysidine or (2) possible changes in host modified nucleosides.

In conclusion, our study has revealed distinct release dynamics of modified nucleosides within bacterial systems, which deviate significantly from their mammalian counterparts. Most modified nucleosides were not released under normal growth conditions for *E. coli*. Instead, m^2^A was readily released upon cell lysis by antibiotics or by immune cell exposure. Lastly, our clinical observations revealed elevation of m^2^A levels in the urine of bacterial infection patients. This research serves as the basis to understand bacterial modified nucleoside metabolism and its potential use in the clinic.

## SUPPLEMENTAL MATERIAL

Supplemental Figures and Tables mbio.03124-24-s0001.pdf Fig. S1 to S7 and Tables S1 to S5.

ASM does not own the copyrights to Supplemental Material that may be linked to, or accessed through, an article. The authors have granted ASM a non-exclusive, world-wide license to publish the Supplemental Material files. Please contact the corresponding author directly for reuse.
