# Supplementary material for: Bacteria-specific modified nucleoside is released and elevated in urine of patients with bacterial infections
Source: mBio. 2024 Dec 11;16(1):e03124-24. doi: 10.1128/mbio.03124-24 (PMC11708014; doi:10.1128/mbio.03124-24)
Supplement: Supplemental Figures and Tables — Fig. S1 to S7 and Tables S1 to S5. [file mbio.03124-24-s0001.pdf]

Nucleosides	Precursor ion (m/z)	Product ion (m/z)
A	268.0000	136.0000
U	245.0000	113.0000
G	284.0000	152.0500
C	244.2000	112.1000
mcmo^{5}U	333.1000	201.1000
ms^{2}i^{6}A	382.2000	182.0000
s^{4}U	261.0500	129.0000
s^{2}C	260.2000	128.2000
m^{4}Cm	272.2000	126.2000
m$^{4}_{2}$Cm	286.3000	140.3000
m^{2}A	282.3000	150.0500
m^{6}t^{6}A	427.3000	295.3000
m$^{4}_{2}$C	272.2000	140.0000
Ψ	245.1000	209.0000
D	247.0000	115.0000
I	269.1000	137.1000
mnm^{5}s^{2}U	304.1000	172.0000
Um	259.0000	113.0000
Cm	258.2500	112.0500
Gm	298.1000	152.1000
Am	282.1000	136.0000
m^{1}A	282.3000	150.0500
m^{6}A	282.3000	150.0500
m^{1}G	298.0000	166.0000
m^{2}G	298.0000	166.0000
m^{7}G	298.0000	166.0000
ms^{2}t^{6}A	459.1000	327.1000
m^{5}U	259.1000	127.0000
m^{5}C	258.0000	126.0000
t^{6}A	413.1000	281.1000
m$^{2}_{2}$G	312.0000	180.0000
m$^{6}_{2}$A	296.0000	164.0000
ac^{4}C	286.2000	154.0000
i^{6}A	336.2000	204.0000
s^{2}U	261.0500	129.0000

Supplementary Table 1: MRM parameter of nucleosides.

Abbreviation	Molecule Name	Vender	Number
Ψ	pseudouridine	Wako	353-43041
D	dihydrouridine	MCE	HY-113047
mnm^5s^2U	5-methylaminomethyl-2-thiouridine	BIOSYNTH	NM159475
m^5U	5-methyluridine	Wako	320-56601
m^5C	5-methylcytidine	MCE	HY-113135
I	inosine	Wako	099-00231
Cm	2'-*O*-methylcytidine	TCI	M2317
m^7G	7-methylguanosine	SIGMA	M0627
m^1G	1-methylguanosine	CAYMAN	31737
m^2G	N^2-methylguanosine	SCB	sc-215517
m^1A	1-methyladenosine	CAYMAN	16937
m^2A	2-methyladenosine	SCB	sc-500888
m^6A	N^6-methyladenosine	CAYMAN	28833
Um	2'-*O*-methyluridine	TCI	M2290
Gm	2'-*O*-methylguanosine	TCI	M2318
ac^4C	N^4-acetylcytidine	COMBI	QB-9019
m^2_2G	N^2,N^2-dimethylguanosine	MCE	HY-113137
t^6A	N^6-threonylcarbamoyladenosine	SCB	sc-286478
Am	2'-*O*-methyladenosine	MCE	HY-W011552
ms^2t^6A	2-methylthio-N^6-threonylcarbamoyladenosine	TRC	M330590
m^6_2A	N^6,N^6-dimethyladenosine	MCE	HY-101984
i^6A	N^6-isopentenyladenosine	CAYMAN	20522
ms^2i^6A	2-methylthio-N^6-isopentenyladenosine	SCB	SC-484230
m^6t^6A	N^6-methyl-N^6-threonylcarbamoyladenosine	TRC	M696255
s^2C	2-thiocytidine	SCB	sc-283290
m^4Cm	N^4,2'-*O*-dimethylcytidine	BIOSYNTH	NM144592
m^4_2Cm	N^4,N^4,2'-*O*-trimethylcytidine	BIOSYNTH	FT144591
$mcmo^5U$	5-methoxycarbonylmethoxyuridine	BIOSYNTH	NU159494
m^4_2C	N^4,N^4-dimethylcytidine	BIOSYNTH	ND159405
s^2U	2-thiouridine	SCB	SC-220766
s^4U	4-thiouridine	Sigma-Aldrich	T4509
A	adenosine	Wako	015-24591
U	uridine	Wako	213-00771
C	cytidine	TCI	C0522
G	guanosine	TCI	G0171

Supplementary Table 2: Lists of standard for nucleosides.

Target gene for siRNA	Target Sequence	Vender	Code
SLC29A1 #1	GGAACTCTCTCAGTGCCATCTTCAA	Thermo Fisher Scientific	HSS103255
SLC29A1 #2	GAGACCAAGTTGGACCTCA	Thermo Fisher Scientific	s4692
SLC29A1 #3	GATCGTGCTCATTAATTCA	Thermo Fisher Scientific	s4694
SLC29A2 #1	GAAAGTAGCTCTGACCCTGGATCTT	Thermo Fisher Scientific	HSS104895
SLC29A2 #2	GCTCTTTGCCGTTTCTAAT	Thermo Fisher Scientific	s6707
SLC29A2 #3	ACCTGAAGTTTGCCCGCTA	Thermo Fisher Scientific	s6708
SLC29A3 #1	CCCTCACTACCTTCCTCCTGTACAA	Thermo Fisher Scientific	HSS124354
SLC29A3 #2	GCCCTAGAGTTATTACAAA	Thermo Fisher Scientific	s30708
SLC29A3 #3	CCAACATCGAGTCCCTCAA	Thermo Fisher Scientific	s30709
SLC29A4 #1	CCACCTTCAGAGCCCTGTTACTGCA	Thermo Fisher Scientific	HSS153753
SLC29A4 #2	CGGGCGTAGTGATGAGCTT	Thermo Fisher Scientific	s48255
SLC29A4 #3	GCCGTGACCTACTTCATCA	Thermo Fisher Scientific	s48256

Gene name	Forward	Reverse
18S rRNA	GTAACCCGTTGAACCCCATT	CCATCCAATCGGTAGTAGCG
TNF-α	CCTCTCTCTAATCAGCCCTCTG	GAGGACCTGGGAGTAGATGAG
IL-6	ACTCACCTCTTCAGAACGAATTG	CCATCTTTGGAAGGTTCAGGTTG
IL-15	CAGCCCATACAAGATCGTATTG	GCCACGGTAAATCCTTAAGTATTGAA
SLC29A1	TGAGCGGAACTCTCTCAGTG	TGAGGTAGGTGAATAACAGCAGG
SLC29A2	TCAGTGCAGTCCTACAGGG	GGCGTGATAAAGTACCCCAGG
SLC29A3	TTGAGAGCTACCTTGCCGTTG	CAGTGCAGTTATCACCATGAAGA
SLC29A4	GCTTTCACGGATACTACATTGGA	ATGTCAAACACGATGGAGGTC

Supplementary Table 3: List of siRNAs and primers used for RT-PCR in this study.

	**Bacterial Infection(N=7)**		**Non-Bacterial Infection(N=9)**	
**Age** Median, (Range)	72 (81-30)		65 (79-47)	
**Sex**				
**Male(n, %)** **Female(n, %)**	0(0) 7(100)		3(33.3) 6(66.7)	
**Race**				
**East Asian(%)**	100%		100%	
	**Normal Phase**	**Fever Phase**	**Normal Phase**	**Fever Phase**
**CRP(mg/dL)** Median, (Range)	0.16 (2.54-0.06)	1.34 (17.2-0.32)	0.22 (2.32-0.06)	2.02 (12.94-0.11)
**WBC(10^3/mL)** Median, (Range)	7.5 (8.8-2.0)	7.9 (10.6-2.1)	8.0 (12.3-2.8)	6.7 (16.2-2.5)
**Urine Creatinine (mg/dL)** Median, (Range)	43.06 (63.2-25.4)	45.82 (80-19.1)	47.8 (132.5-29.4)	71.2 (316.9-25.9)
**Urine Urea Nitrogen (mg/dL)** Median, (Range)	233.3 (266.5-118.1)	91.9 (163.5-11.3)	341.3 (718.6-98.7)	323.6 (545.2-122)
**Urine Na (mmol/L)** Median, (Range)	66 (103-38)	86 (117-16)	98 (136-10)	66 (210-16)
**Urine K (mmol/L)** Median, (Range)	16 (45.9-6)	10.8 (31-8)	19 (42-8)	19.9 (43-5)
**Urine Cl (mmol/L)** Median, (Range)	56 (99-33)	84 (108-15)	83 (148-15)	59 (166-20)
**Cause of Fever** n, (%)	Catheter Infections(3, 42.8) Bacterial Pneumoniae (2, 28.6) Urinary Tract Infection(2, 28.6)		Autoimmune Diseases(4, 44.4) Allergic Diseases(3, 33.3) Virus Infection(2, 22.2)	

Supplementary Table 4: Clinical information pertaining to bacterial infection patients and non-bacterial infection patients.

	Healthy volunteer(N=10)	Bacterial Infection(N=16)
**Age**<br>Median, (Range)	35<br>(39-29)	73<br>(85-57)
**Sex**		
**Male(n, %)**<br>**Female(n, %)**	10(100)<br>0(0)	11(68.8)<br>5(31.2)
**Race**		
**East Asian(%)**	100%	100%
**CRP(mg/dL)**<br>Median, (Range)	-	9.975<br>(23.02-0.14)
**WBC(10^3/mL)**<br>Median, (Range)	-<br>-	9.8<br>(26.9-1.2)
**Urine Creatinine (mg/dL)**<br>Median, (Range)	211.45<br>(553.9-38.1)	92.2<br>(217.5-22.9)
**Urine Urea Nitrogen (mg/dL)**<br>Median, (Range)	1179.4<br>(1543.5-311.7)	665.5<br>(1275.2-154.5)
**Urine Na (mmol/L)**<br>Median, (Range)	145.5<br>(324-58)	74<br>(187-15)
**Urine K (mmol/L)**<br>Median, (Range)	56.5<br>(121.1-13.8)	42.1<br>(79.2-9)
**Urine Cl (mmol/L)**<br>Median, (Range)	183.5<br>(271-57)	69<br>(194-15)
**Cause of Fever**<br>n, (%)	-	Bacterial Pneumoniae (7, 43.8)<br>Catheter Infections(4, 25)<br>Cholecystitis(3, 18.8)<br>Urinary Tract Infection(1, 6.2)<br>Cellulitis(1, 6.2)

Supplementary Table 5: Clinical information pertaining to healthy volunteers and bacterial infection patients.

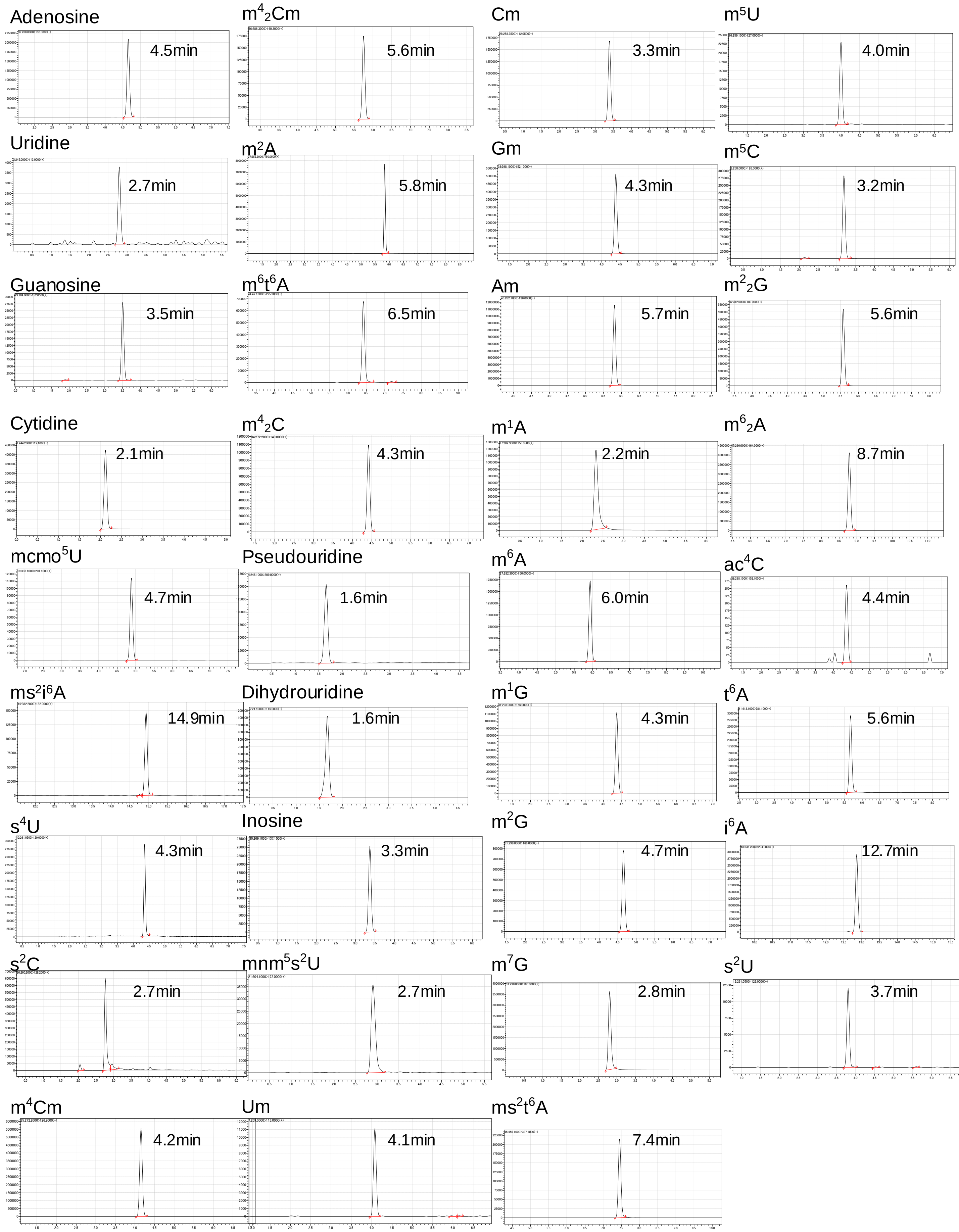

Supplementary Figure 1: Standard peaks of nucleosides observed by LC-MS. Abbreviations are the same as in Figure 1A.

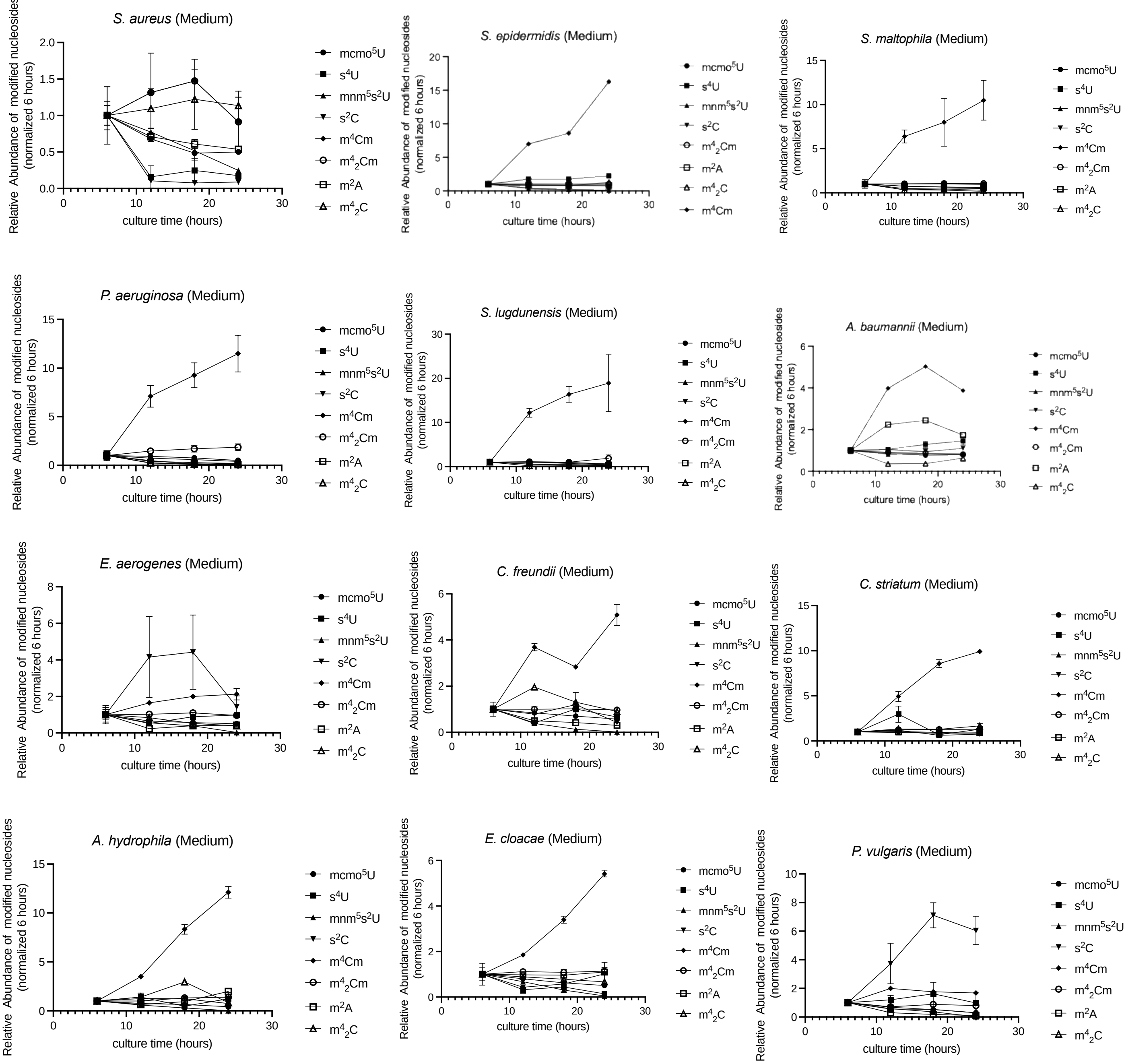

Supplementary Figure 2: Chart analysis of modified nucleoside levels in the culture medium from each bacterial species over time. Abbreviations refer to Figure 1A.

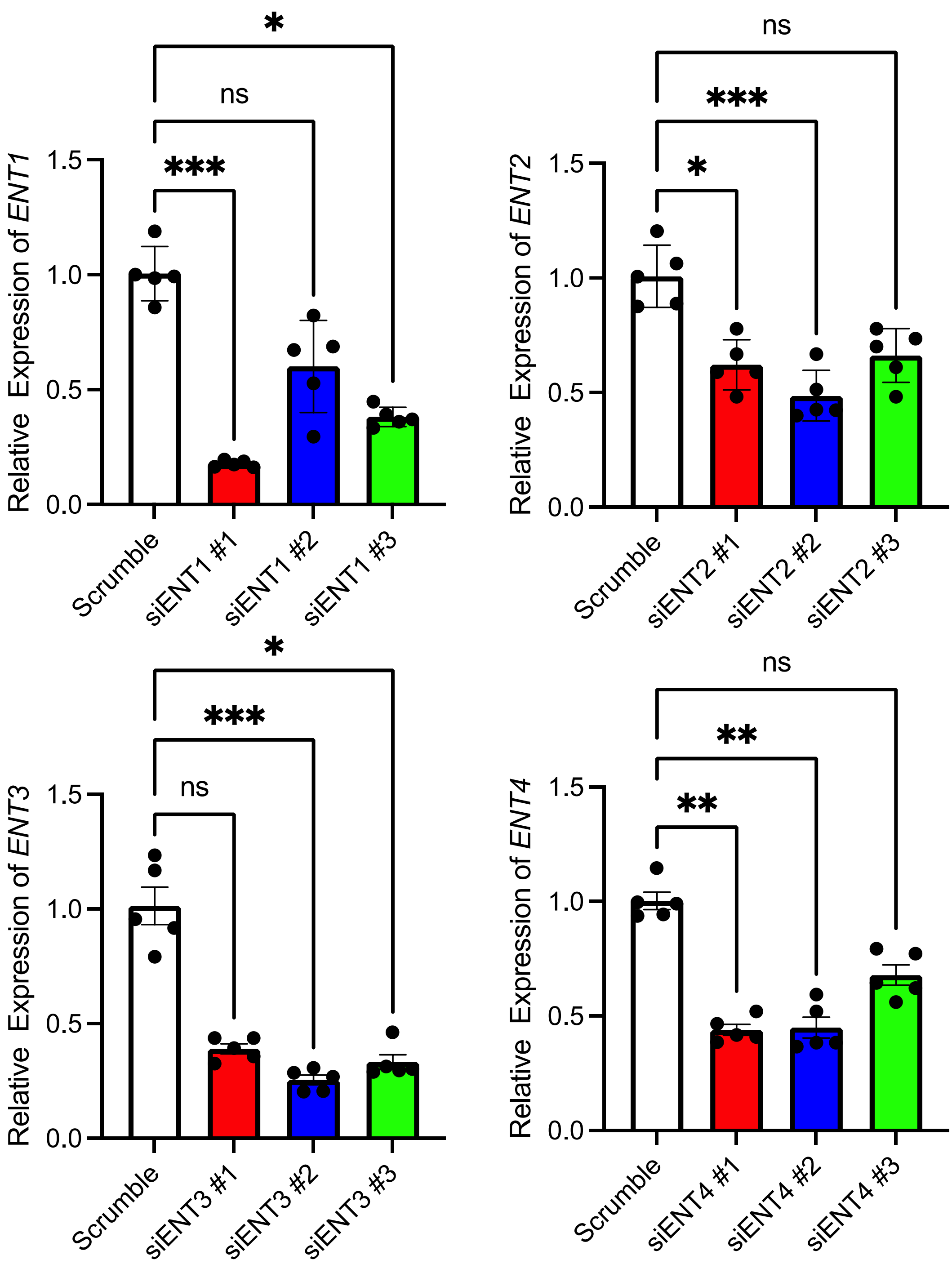

Supplementary Figure 3: RT-PCR analysis following knockdown of ENTs using siRNA. *: $p < 0.05$, **: $p < 0.01$, ***: $p < 0.001$. Data were analyzed using a Kruskal–Wallis test and Dunn's multiple comparison test.

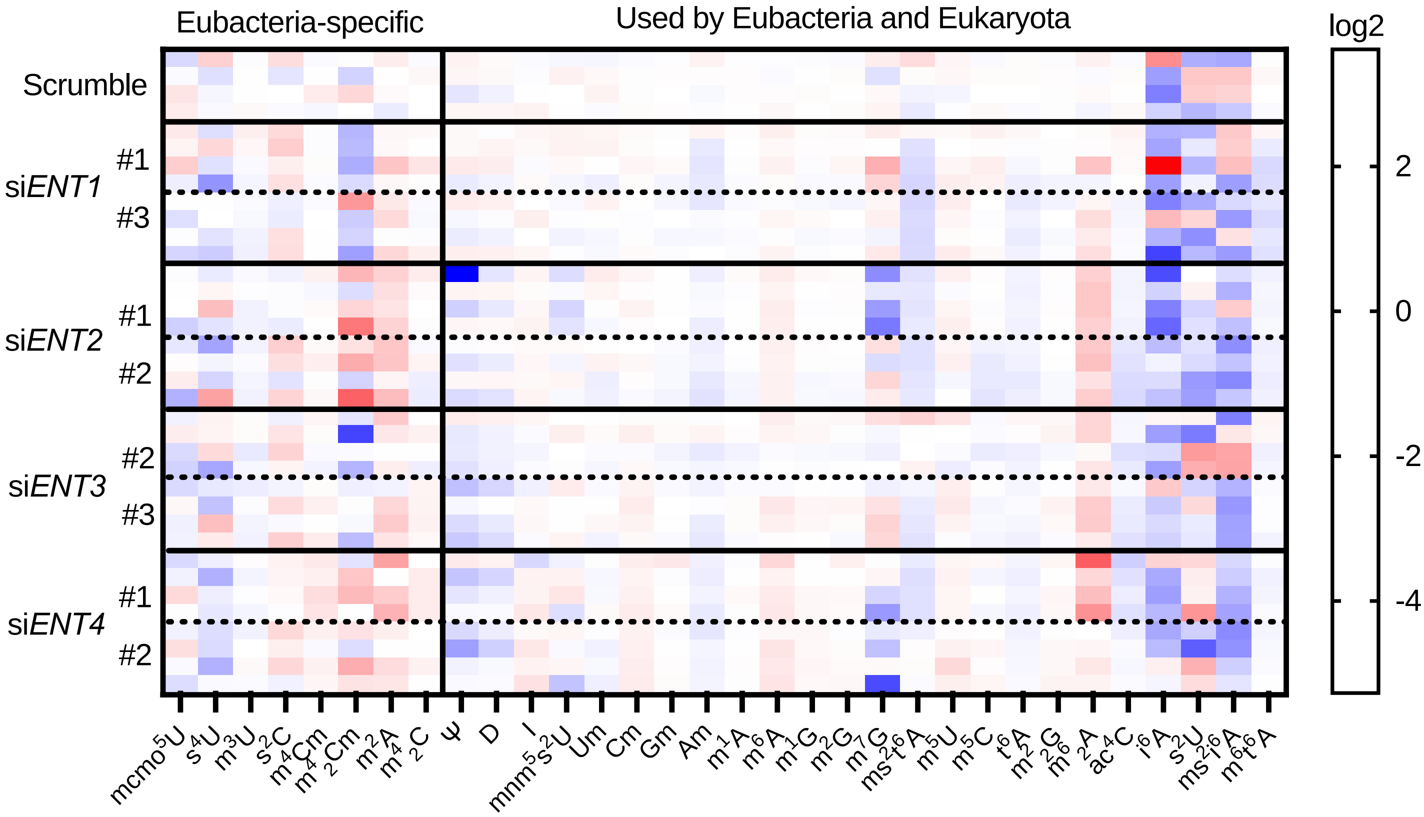

Supplementary Figure 4: Heatmap analysis of modified nucleoside levels in culture medium of *E. coli* (ATCC25922) after knockdown of ENTs using siRNA. The color scale indicates the auto-scaled relative mean of four biological replicates. Abbreviations in the heatmap refer to Figure 1A.

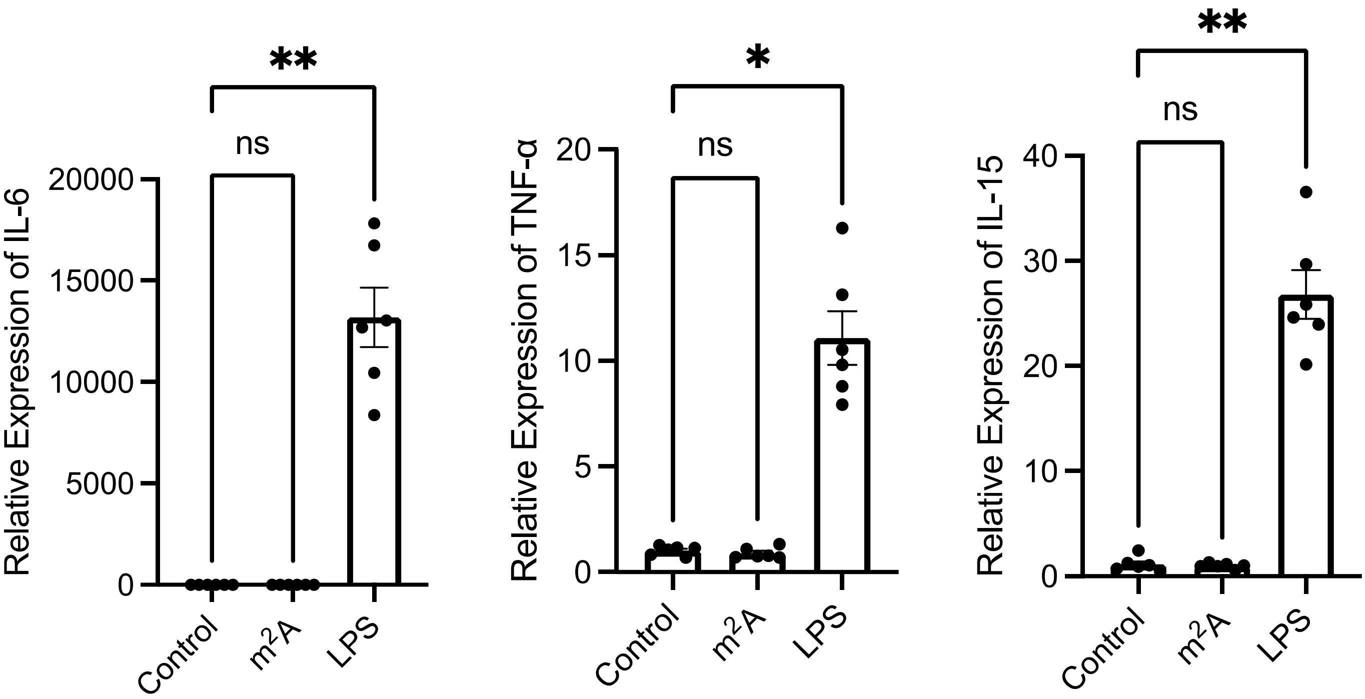

Supplementary Figure 5: RT-PCR analysis following administration of m²A. *: $p < 0.05$, **: $p < 0.01$. Data were analyzed using the Kruskal–Wallis test and Dunn's multiple comparison test.

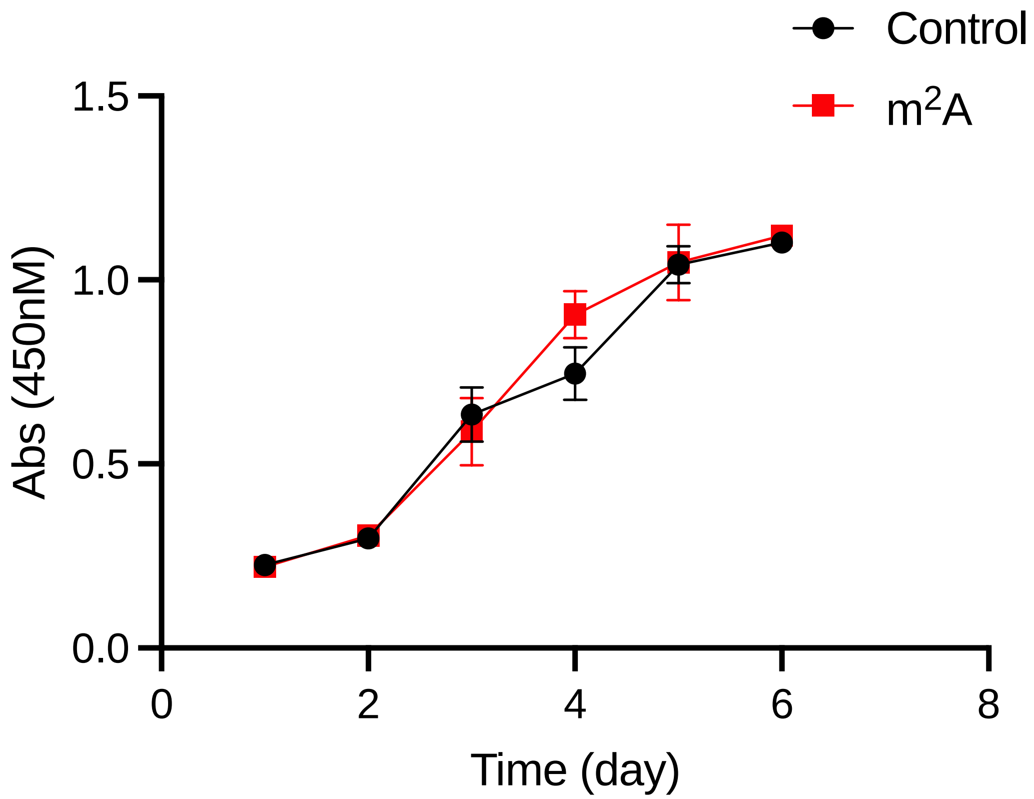

Supplementary Figure 6: Cell growth following treatment of HeLa cells with m^{2}A.

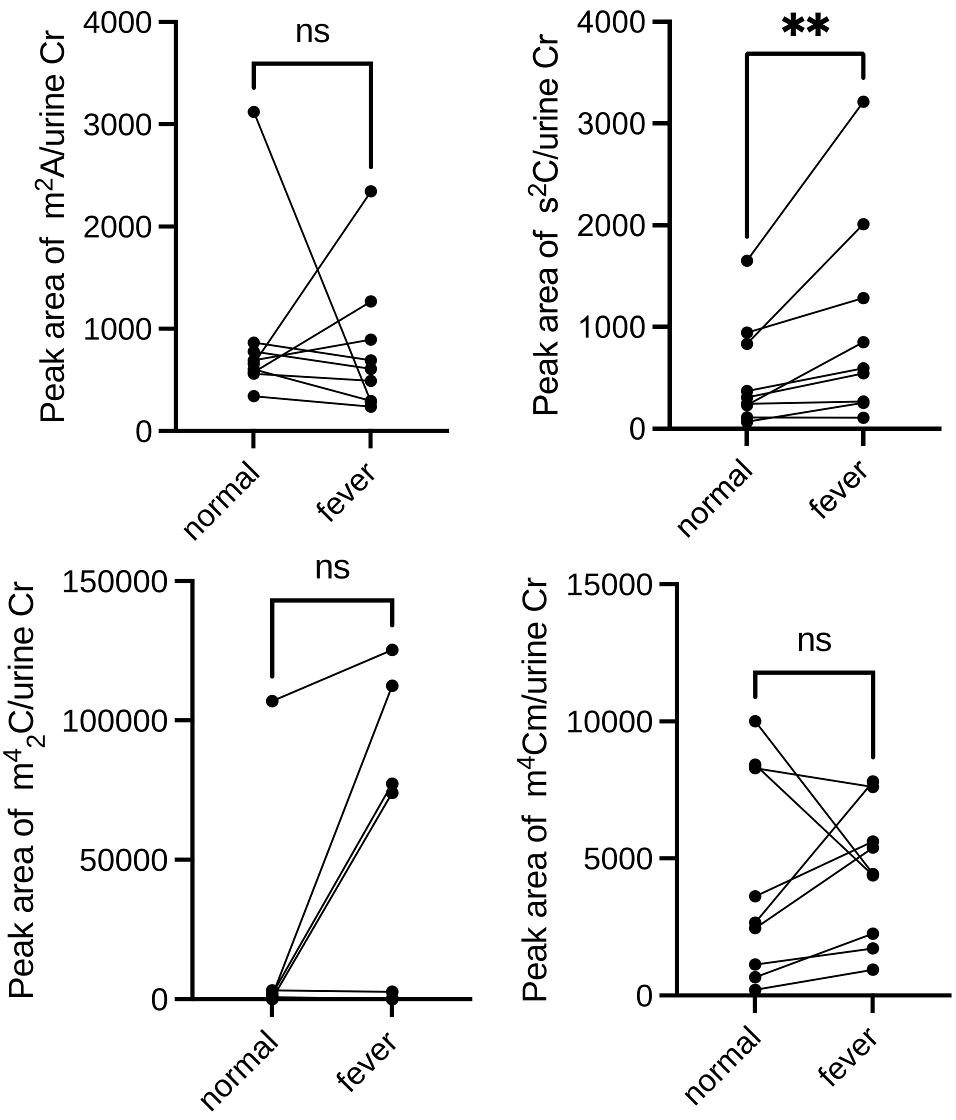

Supplementary Figure 7: Measurements of bacteria-specific modified nucleosides in the urine of patients with non-bacterial infection at the normal phase and fever phase. LC-MS peak areas of modified nucleosides divided by urine creatinine are shown. **: $p < 0.01$. Data were analyzed using a Wilcoxon signed-rank test.
